# Emerging therapies for cartilage regeneration in currently excluded ‘red knee’ populations

**DOI:** 10.1038/s41536-019-0074-7

**Published:** 2019-05-30

**Authors:** Anthony R. Martín, Jay M. Patel, Hannah M. Zlotnick, James L. Carey, Robert L. Mauck

**Affiliations:** 1 McKay Orthopaedic Research Laboratory, Department of Orthopaedic Surgery, Perelman School of Medicine, University of Pennsylvania, Philadelphia, PA 19104 USA; 2 Translational Musculoskeletal Research Center, Corporal Michael J. Crescenz Veterans Affairs Medical Center, Philadelphia, PA 19104 USA; 3 Department of Bioengineering, School of Engineering and Applied Science, University of Pennsylvania, Philadelphia, PA 19104 USA

**Keywords:** Mesenchymal stem cells, Osteoarthritis

## Abstract

The field of articular cartilage repair has made significant advances in recent decades; yet current therapies are generally not evaluated or tested, at the time of pivotal trial, in patients with a variety of common comorbidities. To that end, we systematically reviewed cartilage repair clinical trials to identify common exclusion criteria and reviewed the literature to identify emerging regenerative approaches that are poised to overcome these current exclusion criteria. The term “knee cartilage repair” was searched on clinicaltrials.gov. Of the 60 trials identified on initial search, 33 were further examined to extract exclusion criteria. Criteria excluded by more than half of the trials were identified in order to focus discussion on emerging regenerative strategies that might address these concerns. These criteria included age (<18 or >55 years old), small defects (<1 cm^2^), large defects (>8 cm^2^), multiple defect (>2 lesions), BMI >35, meniscectomy (>50%), bilateral knee pathology, ligamentous instability, arthritis, malalignment, prior repair, kissing lesions, neurologic disease of lower extremities, inflammation, infection, endocrine or metabolic disease, drug or alcohol abuse, pregnancy, and history of cancer. Finally, we describe emerging tissue engineering and regenerative approaches that might foster cartilage repair in these challenging environments. The identified criteria exclude a majority of the affected population from treatment, and thus greater focus must be placed on these emerging cartilage regeneration techniques to treat patients with the challenging “red knee”.

## Introduction

Articular cartilage injuries pose a significant clinical challenge in orthopaedics. The high prevalence of injury and lack of intrinsic tissue healing capacity leave a relatively young and healthy population on the path to degenerative osteoarthritis (OA) without intervention.^1^ Arthroscopic procedures reveal the presence of chondral lesions in greater than 60% of patients,^2,3^ and yearly incidence rates of chondral lesions nearly tripled between 1996 and 2011.^4^ The most common arthroscopic intervention for chondral injuries is chondroplasty, or removal of the loose pieces of cartilage. While this provides short-term symptomatic relief, the remaining cartilage is more susceptible to wear and accelerated degeneration. Another common intervention is microfracture, which involves penetrating the subchondral bone to allow bone marrow to fill the defect. This approach results in the formation of mechanically-inferior fibrocartilage tissue.^5,6^ Mosaicplasty, typically osteochondral autologous transplantation (OATs), is an open procedure where osteochondral plugs are harvested from non-weight bearing areas and transferred into the defect. However, this transplanted cartilage can cause donor site morbidity, does not integrate well with the existing cartilage, and has been shown to degenerate over the long term.^7^

More modern cartilage regeneration approaches include autologous chondrocyte implantation (ACI) and now matrix-associated autologous chondrocyte implantation (MACI), as well as autologous matrix-induced chondrogenesis (AMIC). MACI is a two-stage procedure (and an advance on the original ACI procedure), where healthy cartilage cells are harvested from the patient, expanded, seeded in a collagen matrix, and then re-implanted into the cartilage defect. AMIC, on the other hand, is a single-stage procedure where a cell-free collagen matrix is implanted into a cartilage defect in combination with microfracture. These treatments have demonstrated improved outcomes compared to traditional techniques (chondroplasty/microfracture); however, they are mostly restricted in their application and evaluation to a small fraction of the patient population with near-ideal surgical conditions.^1^ This sub-population of patients (with “green knees”) presents the highest probability of successful resolution of symptoms following intervention, and so are most often included in initial clinical trials. These patients are differentiated from those having “red knees”, whose cartilage pathologies are more severe or whose co-morbidities preclude them from these cartilage repair procedures.^8^ This “red knee” cohort is typically only left with short-term palliative treatment options, such as oral non-steroidal anti-inflammatory drugs (NSAIDs) or intra-articular injections of corticosteroids or hyaluronic acid. While these treatments do provide temporary inflammation and pain relief, bioactive factors reside in the joint for <2 months, necessitating frequent injections.^9^ Lacking available options for long-term repair and/or regeneration, these “red knee” patients are often neglected and almost certainly destined for total joint replacement.

To address this topic in a systematic fashion, this review first analyzes the disease burden of articular cartilage injuries, focusing on those currently deemed treatable by repair and restoration procedures. Next, we examine clinical trial exclusion criteria for cartilage lesion characteristics and patient co-morbidities that identify a “red knee” which is contra-indicated for treatment using current technologies. Patient attributes that result in exclusion by >50% of trials are used to inform the topics/conditions that should be addressed in regenerative strategies addressing a broader patient population. We then highlight recent advances in pre-clinical and translational regenerative technologies that may address these challenges in cartilage restoration, and identify areas with the most pressing need for continued development.

## Disease burden and treatment trends

Cartilage injuries are extremely common, and lesions are often present in asymptomatic patients. For instance, Curl et al.^2^ reported that 63% (19,827/31,516) of knee arthroscopies for any indication identified chondral lesions, 32% of which had exposed bone, categorizing them as grade IV on the Outerbridge scale. Widuchowski et al.^3^ supported this high prevalence, reporting chondral lesions in 15,074 of 25,124 arthroscopies (60%). Of these, 9% of patients had Grade III or IV chondral lesions and were under 50 years old, meeting conservative indications for cartilage repair surgery. In both studies, the authors found that concomitant ligamentous or meniscal pathology was present in ~70% of cases, corresponding to the high prevalence of traumatic etiology in these patients. The disease burden of cartilage injuries is also growing, with the annual incidence of cartilage injuries increasing from 22/100,000 in 1996 to 61/100,000 in 2011, across all age groups and sexes. The percent of cartilage injuries treated ranged from 13.8 to 22.1% during this time period, yet only 1% of these repair procedures involved advanced techniques such as chondrocyte transplantation.^4^

**Fig. 1:**
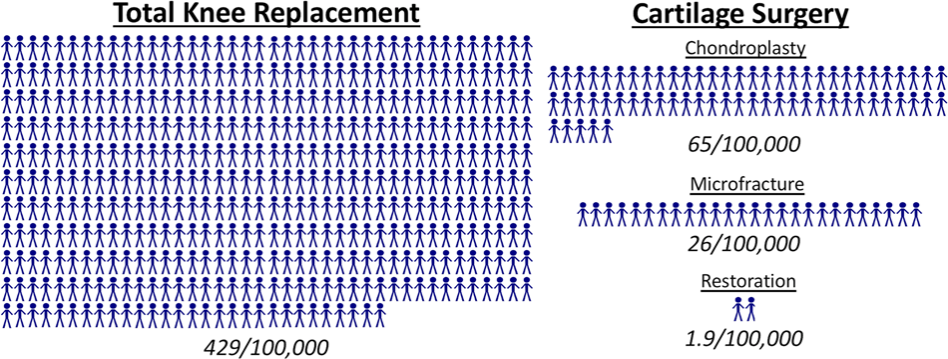
Yearly incidence rate of primary total knee replacement^11^ and cartilage surgeries including chondroplasty, microfracture, and restoration (ACI, MACI, AMIC).^10^ Data presented as incidence per 100,000 persons

Another study based on 25 million privately insured patients in the United States found that the yearly incidence rate of cartilage repair surgery increased from 63/100,000 in 2006 to 93/100,000 in 2011. In 2011, 70% of the procedures involved chondroplasty (smoothing the defect by removing loose strands of cartilage), 28% involved microfracture or subchondral drilling (techniques used to release marrow elements), and only 2% involved the use of advanced cartilage restoration techniques (osteochondral autograft transfer, ACI).^10^ In contrast, primary total knee arthroplasty (TKA) had an incidence rate of 429/100,000,^11^ and recent projections estimate an 85% increase in TKAs by 2030.^12^ The large disparity between the incidence of TKA and that of cartilage repair surgeries (Fig. 1) suggests a pressing need for more advanced cartilage repair technologies that might obviate the need for TKA and control the high socio-economic burden of end-stage knee arthritis.

Cartilage restoration procedures may also provide financial benefits for both patients and healthcare systems. A recent cost-benefit analysis with at least 5-year follow-up estimated the direct plus indirect costs of ACI to be $16,781, nearly 20% less than that of TKA ($20,568).^13^ Future savings provided by ACI could include avoiding multiple debridement and microfracture procedures on the same cartilage lesion and the need for a total joint replacement early in life and a subsequent revision TKA. This could circumvent the work-time lost from OA debilitation or post-surgery rehabilitation. The improvement of cartilage restoration technologies and techniques may result in shorter operative times, quicker patient recoveries, and perhaps diminished requirement for a preliminary cartilage harvest procedure, all leading to lower overall costs. Broadening the applicability of these restorative procedures and scaling up the industry of cartilage therapies would only serve to further reduce manufacturing costs, and ultimately, the financial burden on both patients and providers.

## Defining the “red knee”

Several studies have shown improved outcomes when comparing ACI, MACI, and AMIC to microfracture.^14–18^ However, these advanced restorative techniques still present increased failure rates in knees with common comorbidities (larger lesion size, “kissing lesions” on opposing articular surfaces, and pre-existing arthritic changes).^19^ One study evaluating MACI with long-term follow-up (5–12 years) found failure rates of 18.2 and 87.5% in complex and salvage cases, respectively, compared to a failure rate of only 4.3% in less complex cases.^20^ Similarly, Filardo and colleagues found that MACI treatment in osteoarthritic knees had a failure rate of 27.3% at 9-year follow-up.^21^ Treatment failure correlated with degenerative lesion origin, longer symptom duration, larger lesion size, older age, and prior knee surgery.^22^

**Fig. 2:**
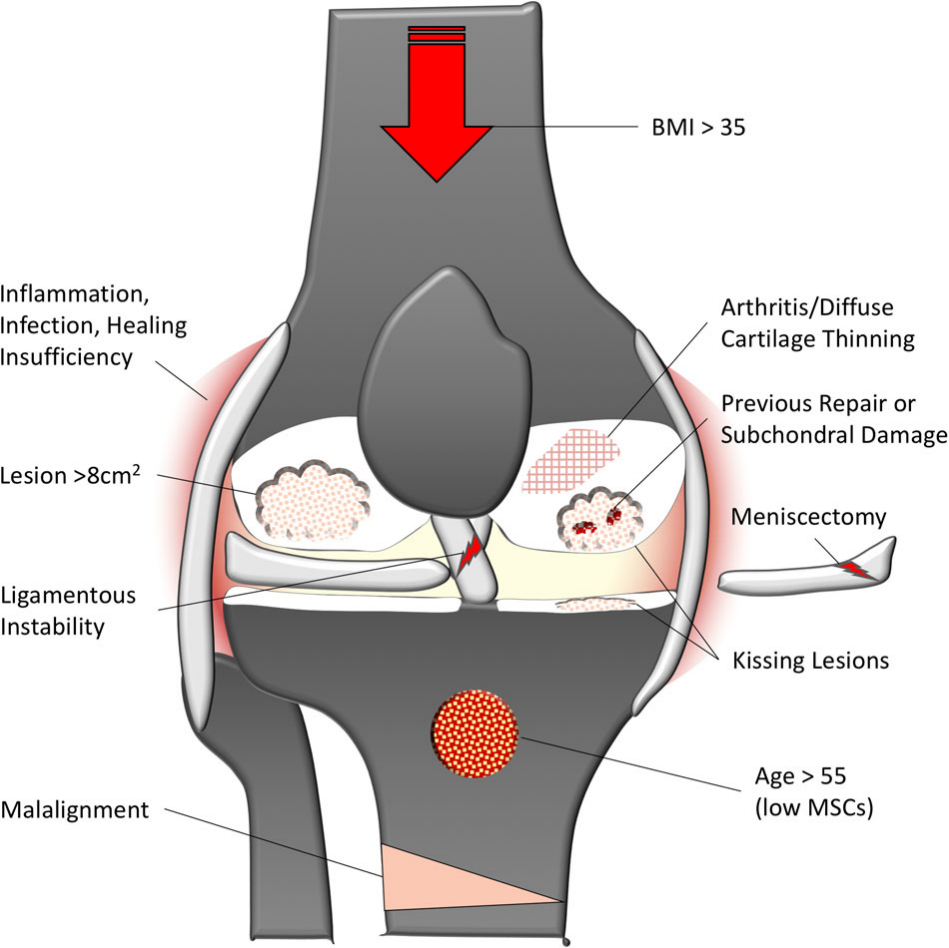
Illustration of common exclusion criteria that define the “red knee”

These case reports and series suggest optimal conditions for repair (i.e., ‘green knees’), and conditions in which repair is likely to be unsuccessful (i.e., ‘red knees’). This is often due to circumstances other than the cartilage defect or procedure itself. These concepts have influenced the manner in which new technologies are evaluated in clinical trials, where conditions most conducive to a successful therapy (i.e., ‘green knees’) are selected for initial trials in humans. To better understand these factors defining the ‘red knee’, we conducted a systematic review of exclusion and inclusion criteria for cartilage restoration clinical trials on “clinicaltrials.gov” using the search term “knee cartilage repair” on 1 January 2019. Studies that had been suspended, terminated, or withdrawn, as well as studies of unknown status, were excluded from the search, leaving 60 studies for review. Follow-up studies and studies using only intra-articular injections were excluded to focus on new cartilage restoration procedures. This yielded 33 clinical trials (Supplemental Table) that were used to extract inclusion and exclusion criteria for tabulation (Supplemental Methods). Common exclusion criteria are illustrated schematically in Fig. 2.

**Fig. 3:**
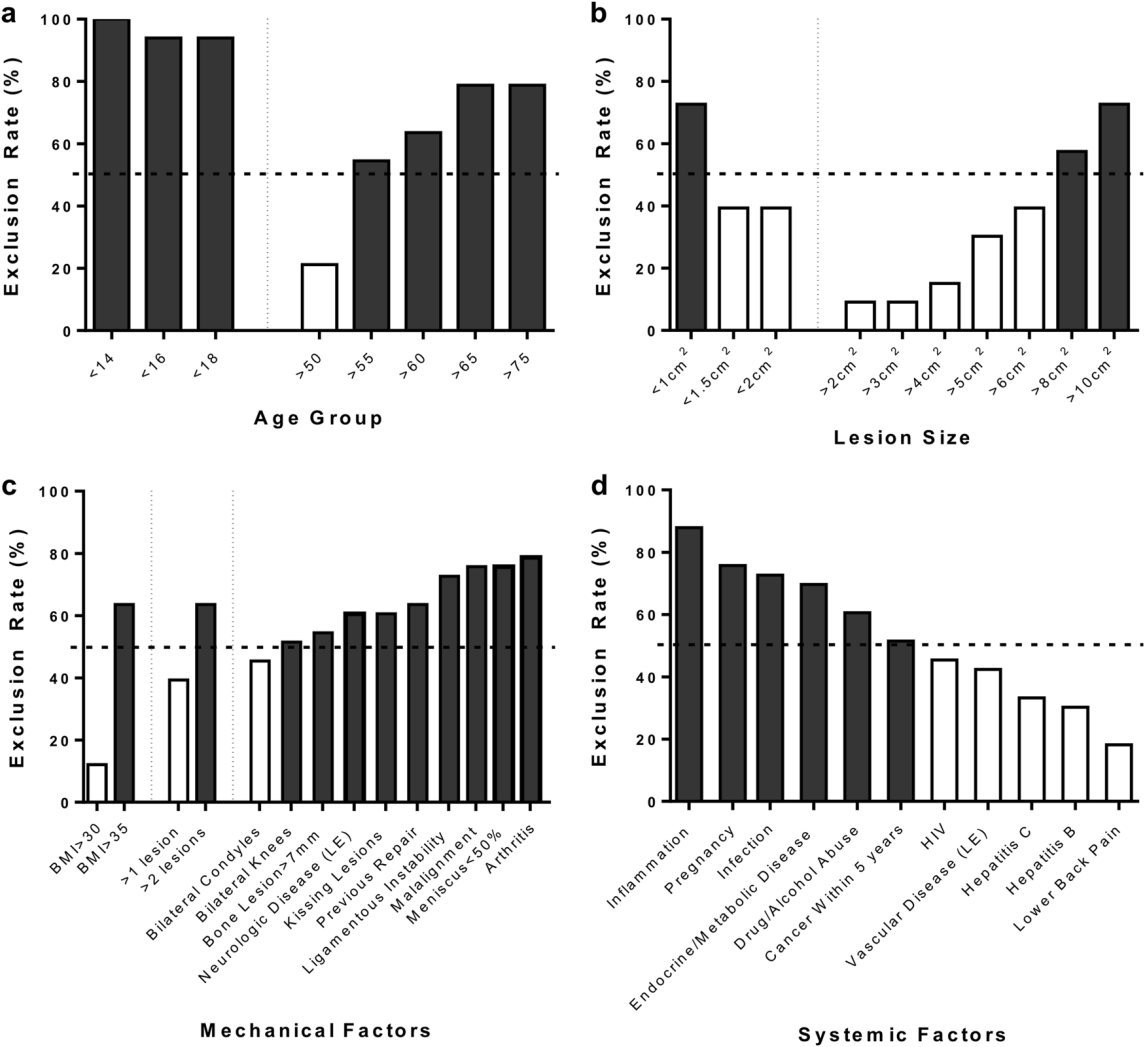
Graphical display of the percentage of clinical trials excluding patients with regards to **a** age group **b** lesion size, **c** mechanical comorbidities, and **d** common systemic comorbidities. Data presented as a percentage, *n* = 33 studies analyzed. Dashed line represents 50% exclusion cutoff for defining the “red knee” population. Exclusion criteria above this threshold are presented with black bars, and criteria below this threshold are presented with white bars. LE lower extremity; BMI body mass index

Patient age was the most common exclusion criteria used in trials, with over half the trials excluding patients over the age of 55 or under the age of 18 (Fig. 3a). The number of chondrocytes and bone marrow-derived MSCs, as well as their proliferative and matrix forming potential, may decline in adulthood.^23–25^ This might limit the healing capacity of cartilage in older patients, and thus is a common exclusion criterion. The lower limit of patient age exclusion relates to the legal age of adults capable of signing informed consent to participate in the trial in the United States. Trials in Europe were more likely to include patients in the 14–17 age range.

Lesion size was another common exclusion criterion, with 58% of clinical trials excluding lesions that were >8 cm^2^ in size (Fig. 3b). Similarly, patients with more than two lesions were excluded by 64% of trials, and patients with signs of joint-wide OA were excluded by 79% of trials. Joints requiring a large surface area of cartilage restoration are challenging for a number of reasons, including mechanical demands, graft fixation, and the large number of cells required for transplantation (in cell based therapies). Interestingly, lesions <1 cm^2^ were also excluded by over 50% of trials, likely due to satisfactory outcomes in small lesions treated by microfracture.^26^ Of note, 64% of trials also excluded lesions that had been previously treated with either microfracture or cartilage graft, and 55% of trials excluded bone lesions >7 mm in depth. These lesions might be expected to have progressive cartilage wearing and/or subchondral bone damage that could limit the repair and integration capacity of revision tissue engineering therapies applied to the same location, a controversial topic in the field at present.^22,27,28^

Another subset of exclusion criteria relates to scenarios that place excessive mechanical demands on the repair tissue. Over one half of the clinical trials excluded patients with body mass index (BMI) greater than 35, pathology on bilateral knees, neurologic disease of the lower extremities, the presence of kissing lesions, ligamentous instability, joint malalignment (>5° offset), and prior meniscectomy with <50% of the native meniscus remaining (Fig. 3c). These comorbidities increase the risk of excessive focal or abnormal loading and the likelihood for degeneration, and are unfortunately very common in the United States. For example, 15.5% of adults in the US currently have a BMI > 35.^29^ Ligamentous instability and tears (incidence: 46/100,000 person-years^30^), and meniscectomies (incidence: 224/100,000^31^) further compound the high risk of progression to OA^32^ and increase the likelihood of patient exclusion.

Lastly, we identified a group of systemic comorbidities related to overall health that were commonly used as exclusion criteria (Fig. 3d). Patients with systemic or local inflammatory conditions were excluded by 88% of trials. Active inflammatory conditions would be expected to interfere with any repair process, and could thus limit the efficacy of tissue engineering strategies.^33–36^ Similarly, 73% of trials excluded patients with systemic or local infection. Infection could bring about an inflammatory response or directly infect the repair tissue, compromising viability and functional maturation of implanted constructs.^37,38^ Patients with metabolic or endocrine conditions such as diabetes mellitus, parathyroid disease, and chronic kidney disease were excluded by 70% of trials. These common conditions are known to interfere with the healing process, and would likely decrease the integration and survival of implanted constructs.^39–42^ Similarly, patients with a history of drug or alcohol abuse were excluded by 61% of trials. These patients would also have limited healing capacity, increased inflammation, and may not adhere to the strict post-operative recovery protocol common to these trials.^43–46^ Other notable conditions frequently excluded were a history of cancer within 5 years (52%), immunocompromised state (45%), and vascular disease of the lower extremity (42%).

The systematic analysis found that cartilage lesions complicated by limited progenitor cell activity and healing capacity, large size, high mechanical demands, local and systemic inflammation, or other systemic diseases are often excluded from treatment, likely due to complications and poor outcomes after cartilage restoration procedures in this context. While these exclusion criteria are reasonable when first evaluating a new technology, their prevalence in the general population who might benefit from regenerative cartilage procedures is evident by the large number of patients who progress to total joint replacement. If these contraindications could be mediated by improvements in cartilage therapeutics, then the number of persons benefiting from these technologies would be dramatically increased. We identified several exclusion criteria that were used in over 50% of the surveyed clinical trials (Fig. 3—black bars). The following section focuses on emerging pre-clinical and translational research trends early in development in the field of cartilage tissue engineering and regenerative medicine, with a focus on developing technologies that may address these exclusion criteria and broaden the patient population indicated for treatment with advanced cartilage repair therapies.

## Advances in cartilage tissue engineering and regenerative therapeutics—treating the “red knee”

Aging is a significant concern in the field of cartilage regeneration due to its deleterious effects on stem cell density and activity, and the increase in cellular senescence with age.^47,48^ Cellular senescence can affect the differentiation potential, immunomodulatory abilities, and migratory capabilities of both stem cells and chondrocytes. Thus, older patients with cartilage lesions may require additional treatment measures to enhance stem cell or chondrocyte availability and activity at the lesion site. The MACI technique seeks to address these concerns by expanding autologous chondrocytes and seeding them in media designed to favor differentiation towards mature chondrocytes.^49^ Cell-free scaffolds (AMIC) rely on the patient’s native cells to infiltrate the graft during implantation, and are thus at an even higher risk of failure in older patients. Thus, researchers have investigated the use of growth factors to increase cell recruitment, improve cell chondrogenesis, and optimize chondrocyte populations for cartilage regeneration therapies. For example, stromal cell-derived factor 1 alpha (SDF-1α) doubled the recruitment of chondrogenic progenitor cells in a hyaluronate-fibrin hydrogel, which formed hyaline cartilage when cultured in a bovine cartilage explant model.^50^ Local SDF-1α release increased the recruitment of systemically infused bone marrow-derived MSCs by 700% in a mouse model of myocardial infarction (MI),^51^ suggesting that cartilage repair scaffolds releasing SDF-1α may perform synergistically with MSCs from marrow recruitment or intra-articular injections. Similarly, transforming growth factor-beta 3 (TGF-β3) released from cartilage repair scaffolds improved chondrogenesis in vitro and in large animal models.^52,53^ Additional examples of bioactive factors used in pre-clinical and clinical trials are included in Table 1, all of which suggest that tailored growth factor delivery may improve cartilage repair in aged patients.

**Table 1:** Growth factors used to enhance cartilage restoration procedures in recent pre-clinical and clinical trials

Biologic	Delivery method	Delivery model	Results	Study
BMP-2	Oligo(poly(ethylene glycol) fumarate) (OPF) hydrogel with IGF-1 in chondral layer and BMP-2 in bony layer	Rabbit osteochondral defect	Dual delivery of IGF-1 and BMP-2 had a higher proportion of subchondral bone repair, greater bone growth at the defect margins, and lower bone specific surface than the single delivery of IGF-1	Ref. ^132^
BMP-7	Graphene oxide nanoparticles in collagen/chitosan hydrogel	Rat cartilage defect	Hydrogel/GO-np protected cartilage by the Rank/Rankl/OPG signal pathway	Ref. ^133^
	PLGA scaffold with BMP-7/TGF-B3 nanocomplexes	in vitro human MSCs	Controlled supplementation of BMP-7 can improve the chondrogenic effect of TGF-β3, and scaffolds loaded with this combination of growth factors can induce cartilage formation in hMSC cultures	Ref. ^134^
FGF-18 (Sprifermin)	Intraarticular injection	Human knee	qMRI showed increased cartilage thickness in a dose-dependent manner in knee OA patients with acceptable safety profile at 3 years	Ref. ^135^
	Collagen membrane (Chondrogide)	Sheep cartilage defect	Potentiated the healing of a microfracture treated cartilage defect with improved weight bearing, O’Driscoll sore, and Type II collagen staining	Ref. ^136^
IGF-1	Porous poly(lactic-co-glycolic acid) (PLGA) scaffold	Rabbit proximal tibial growth plate	Regeneration of cartilage, albeit with disorganized structure, at the site of implantation of IGF-I-releasing scaffolds; in contrast, only bone was formed in empty defects and those treated with IGF-free scaffolds	Ref. ^137^
	Peptide hydrogel, heparin-bound	in vitro bovine chondrocytes	Increased sulfated glycosaminoglycan and hydroxyproline content of chondrocyte-seeded hydrogels, Cartilage explants cultured adjacent to functionalized hydrogels had increased proteoglycan synthesis	Ref. ^138^
SDF-1	Hyaluronate-fibrin hydrogel	Bovine cartilage explants	Improved chondrogenic progenitor cell recruitment and integration strength, mechanical properties similar to native, hyaline histological morphology	Ref. ^50^
	Transduced allogenic hyaline cartilage graft	Mouse subcutaneous	Activation and recruitment of endogenous stem cells in both peripheral blood and within the graft, enhanced chondrogenesis	Ref. ^139^
TGF-B1	Transduced allogenic chondrocytes (Invossa), intraarticular injection	Human knee	Hyaline cartilage regeneration with improved IKDC and VAS scores	Ref. ^140^
TGF-B3	Hyaluronate or PCL nanofibers	Pig cartilage defect	Increased ICRS-II histology scores and Type II collagen staining	Ref. ^52^
	Collagen hydrogel in PCL/hydroxyapatite matrix	Rabbit osteochondral defect	Recruited roughly 130% more cells, uniformly distributed chondrocytes in a matrix with collagen type II and aggrecan, significantly greater thickness, compression and shear properties similar to native cartilage	Ref. ^60^

Another technique to enhance the regenerative capacity of cells is to remove neighboring senescent cells. A recent study by Jeon and colleagues showed that senescent chondrocytes accumulate around traumatic cartilage lesions and are associated with the development of arthritis; clearance of these senescent cells, via intra-articular injection of a senolytic molecule, attenuated the development of arthritis in a mouse ACL transection model.^54^ Another recent study found that rejuvenating aged MSCs with SRT1720, an activator of SIRT1, significantly improved heart function and angiogenesis in a rat MI model compared to control MSCs.^55^ These potential therapeutics targeted towards rejuvenating, optimizing, and recruiting endogenous stem cells will likely increase the efficacy of cartilage tissue engineering techniques in the older patient population.

For patients with a single cartilage lesion greater than 8 cm^2^, multiple lesions, or joint wide cartilage degeneration, whole or hemi-joint tissue engineering is an attractive alternative to metal/plastic joint replacement. These ‘living’ implants could potentially last a lifetime, remodeling in response to applied load and continuously generating new matrix. Recent advances in three dimensional (3D) printing^56^ and rapid prototyping now allow researchers to produce anatomic 3D tissue engineered constructs.^57,58^ Size matching between native and engineered cartilage is critical to maintain joint mobility and function. Computer assisted design (CAD) programs can translate patient scans, via micro-computed tomography (μCT) or magnetic resonance imaging (MRI), into personalized 3D in silico molds.^59^ Using layer-by-layer bioprinting, Mao and colleagues’ generated an entire rabbit humeral head using a poly(ɛ-caprolactone) (PCL) and hydroxyapatite scaffold.^60^ These scaffolds were infused with TGF-β3 to recruit endogenous cells, and inclusion of this growth factor increased cell infiltration into the scaffold by 130%. Another anatomic tissue engineering approach by Moutos and colleagues developed a woven PCL hemispherical scaffold 22 mm in diameter, similarly shaped to the cartilage of the femoral head.^61^ While Mao and colleagues relied on native cells to populate their scaffold for the rabbit shoulder, Moutos and colleagues seeded their hemispherical scaffold with adipose-derived stem cells (ASCs). ASCs in these woven scaffolds released anti-cytokine factors in a sustained manner to reduce joint inflammation, and showed remarkable mechanical features, with tensile, compressive, and shear properties in the native tissue range.^62^ This approach could be particularly useful in cases that a large cartilage surface needs to be replaced, and there are signs of joint inflammation. In a third study, Saxena and colleagues used porcine µCT scans to create negative anatomic molds.^63^ Stem cells encapsulated in a hyaluronic acid hydrogel filled the negative mold, and the resulting tissue was cultured for up to 12 weeks in vitro, exhibiting excellent viability and shape retention. Overall, these studies exemplify how rapid prototyping techniques may be used to generate patient-specific tissue constructs capable of replacing expansive cartilage surfaces.

Other approaches to treat large-scale cartilage defects involve shape-filling chondro-inductive biomaterials. For example, particulated and desiccated allograft tissue, referred to as ‘BioCartilage’,^64^ has been used to repair cartilage in vivo in an equine cartilage large-defect model. A combination of BioCartilage, microfracture, and platelet-rich plasma showed no signs of synovial inflammation, and had superior histological scoring compared to microfracture controls. Others have also utilized devitalized tissue for cartilage repair, including Detamore and colleagues, who showed that stem cells, in the presence of devitalized cartilage microparticles, produced mechanically robust cartilage tissue.^65^ Taken together, there has been continued progress in the field of large-scale cartilage repair, with multiple approaches now being tested in clinically relevant large animal models. Table 2 provides examples of the various fabrication methods discussed. These approaches have the potential to address patients with large lesions that are often excluded from cartilage repair trials.

**Table 2:** Fabrication methods for large cartilage tissue engineering and regeneration therapies in pre-clinical and translational stages

Fabrication Method	Example	Strengths	Weaknesses	Study
Molds	MicroCT and MRI scans used to create custom injection molds for anatomical ovine meniscal cell-seeded alginate meniscus	• Retained native shape through 8 weeks of culture • GAG, Collagen, and Modulus increased with time in culture	• Equilibrium modulus half of native at 8 weeks • Heterogeneous matrix accumulation in center of constructs	Ref. ^57^
	MicroCT scans used to create custom molds for anatomical porcine MSC-seeded hyaluronate hydrogel femoral head cartilage	• Retained native shape through 12 weeks of culture • GAG and dynamic/equilibrium modulus increased with culture time	• Decreased modulus and cell viability at center of constructs • Integration to subchondral bone not addressed	Ref. ^63^
3D Bio-Printing	Extrusion bioprinting of biphasic alginate hydrogels with human chondrocytes and MSCs for osteochondral repair	• Distinctive cartilage-like and bone-like tissue formation seen in respective compartments after 3 weeks in vitro and 6 weeks subcutaneous in immunodeficient mice	• Max compressive modulus ~15 kPa • Limited printing height achieved	Ref. ^141^
	Melt-electrospinning writing of PCL scaffolds infused with gelatin-methacryloyl hydrogel encapsulating human chondrocytes	• Max compressive modulus of 400 kPa with 7% PCL fibers by volume, stress strain curve similar to cartilage • Increased aggrecan and COL1A1 mRNA in compressed constructs	• Cell viability <80% after 7 days in culture • No differences in protein with compression	Ref. ^80^
Woven	Woven PCL hemispherical scaffolds embedded with IL-1Ra lentiviral vector and seeded with human adipose-derived stem cells	• Uniform tissue growth, cartilage biomimetic properties, maintained anatomy after 28 d culture • Robust expression of IL-1Ra prevented MMP activity • Aggregate compressive modulus ~1000 kPa	• Slow scaffold resorption time • High polymer volume occupancy	Ref. ^61^
	Woven aligned collagen threads forming interdigitated arcade structure with macropores filled with MSC pellets, sandwiched between 2 collagen sheets, crosslinked	• Max compressive modulus of 1330 kPa after 28d culture, similar to human cartilage • Excellent fatigue resistance and elastic recoil • Increased GAGs and COL II content with culture time	• Poor integration of pellet with collagen threads • Weave pattern blocks lateral fusion of pellets	Ref. ^142^
Modular	BioCartilage (Arthrex) dessiccated particulated cartilage allograft hydrated with PRP and loaded into defect following microfracture	• Improved cartilage repair histology scores compared to microfracture controls in an equine cartilage defect • Arthroscopic administration, 13 month in vivo results	• Distal lesions showed no improvement • Sclerosis in all defects	Ref. ^64^
	Modular engineered tissue surfaces with self-adhesion of 4 mm agarose gel cylinders with juvenile bovine chondrocytes framed in a custom tibial plateau basket	• Robust bond between modules by 21 days in culture, 3D topography maintained • Compressive modulus and GAG content increase with culture time • No negative impacts with increased total size	• Fibrous tissue at module bonds • Equilibrium modulus ~40–60 kPa	Ref. ^143^

Cartilage restoration procedures have typically avoided small-size defects (<1 cm^2^), likely due to relatively positive outcomes with microfracture in defects of this size.^66,67^ However, the fibrocartilage that forms in these defects is still susceptible to long-term degeneration due to its mechanical insufficiency.^6,68^ Furthermore, treating partial thickness cartilage defects with microfracture may compromise the healthy intact basal layer of cartilage under the defect. Recent biomaterial technologies have been developed to address small full and partial-thickness lesions, with an emphasis on injectable therapeutics for defect-specific filling and ease of application.^69,70^ These injectable scaffolds typically revolve around a material solution that is either photo-polymerized^71,72^ or chemically cross-linked,^73–75^ allowing it to completely fill a defect before solidifying. Combining these scaffold-based injectable formulations with stem cells can also improve the treatment of small focal cartilage injuries. Since cell migration from neighboring cartilage into these small partial-thickness defects is limited,^76^ encapsulating cells within the injected matrix^77,78^ may initiate remodeling and extracellular matrix (ECM) formation immediately, facilitating and improving cartilage regeneration.

**Fig. 4:**
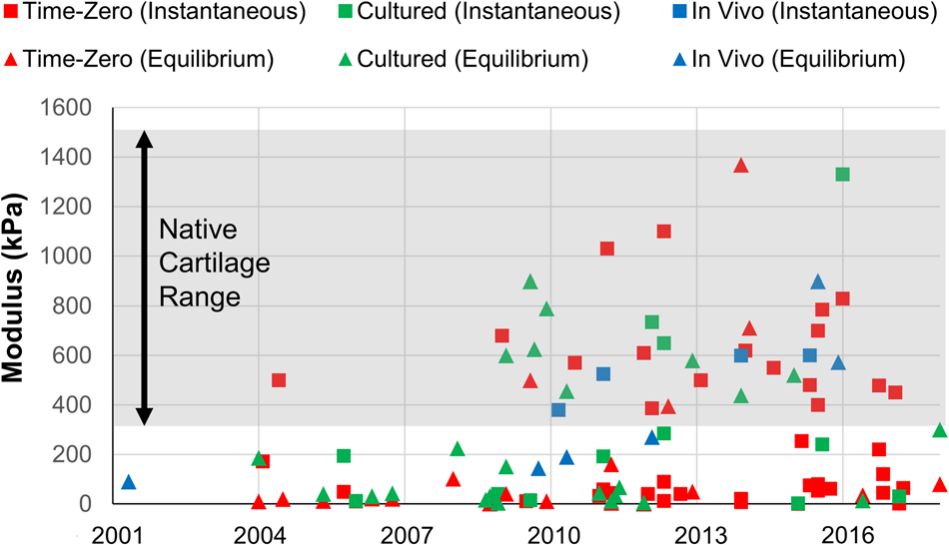
Modulus values (kPa) as a function of time (Jan 2001 to Jan 2018). Squares and triangles represent instantaneous and equilibrium moduli, respectively. Red, green, and blue points represent time-zero scaffold, cultured construct, and mechanical assessments from in vivo studies, respectively. Survey of PubMed literature utilizing search terms “Cartilage”, “Scaffold”, and “Modulus”. Studies with inadequate description of testing methods were excluded

An array of exclusion criteria (BMI > 35, partial meniscectomy, ligamentous instability, malalignment) are related to mechanical comorbidities that current treatments do not address. Due to these mechanical circumstances, the cartilage in these knees is subject to complex stresses that are significantly greater in magnitude than those the healthy “green” knee would experience. Cartilage repair strategies must account for these stresses early in recovery to avoid failure following implantation and patient remobilization. Therapies that rely on scaffold-free cellular approaches, such as ACI, are not designed to handle these mechanical burdens early post-surgery, and thus, utilizing scaffolds with improved bulk mechanical properties could increase efficacy of treatment and promote earlier return to normal activity in these knees. To review the mechanical properties of cartilage scaffolds over the years, a systematic review was performed (Supplemental Methods). Initial scaffold attempts used simple sponges and hydrogels with moduli (both instantaneous and equilibrium) in the tens of kilopascals (kPa), while the modulus of native cartilage is 20–50 times higher. More recently, groups have utilized a variety of fabrication (weaving,^62,79^ 3D printing,^56,80,81^ casting^57,63^) and cross-linking (UV photoinitiator,^82,83^ EDC crosslinking,^83–85^ dehydrothermal treatment^83^) techniques to improve the mechanical properties of the time-zero scaffold (Fig. 4—red squares and triangles). For example, Valonen and colleagues developed a 3D-woven poly(ε-caprolactone) (PCL) scaffold with an aggregate modulus of 550 kPa, well within the range of native articular cartilage.^86^ Other groups have created fiber-reinforced hydrogels, and achieved stiffness values greater than 400 kPa,^80,87^ resulting in replacement scaffolds for defects that require greater mechanical support. An important consideration is balancing the mechanics of these scaffolds with their resorption and ability to form new cartilage tissue.^88,89^ A scaffold should provide architecture and support for initial load-bearing, but exhibit a degradation profile that allows infiltrated cells to respond and form neo-cartilage tissue. Alternatively, in vitro culture of constructs^52,90^ can promote increased deposition and organization of ECM, further elevating mechanical properties of these scaffolds (Fig. 4—green squares and triangles). While more expensive, these lab-grown in vitro engineered replacements have the potential to withstand loadbearing immediately upon implantation, as long as adequate fixation of the grafts can be achieved.

**Fig. 5:**
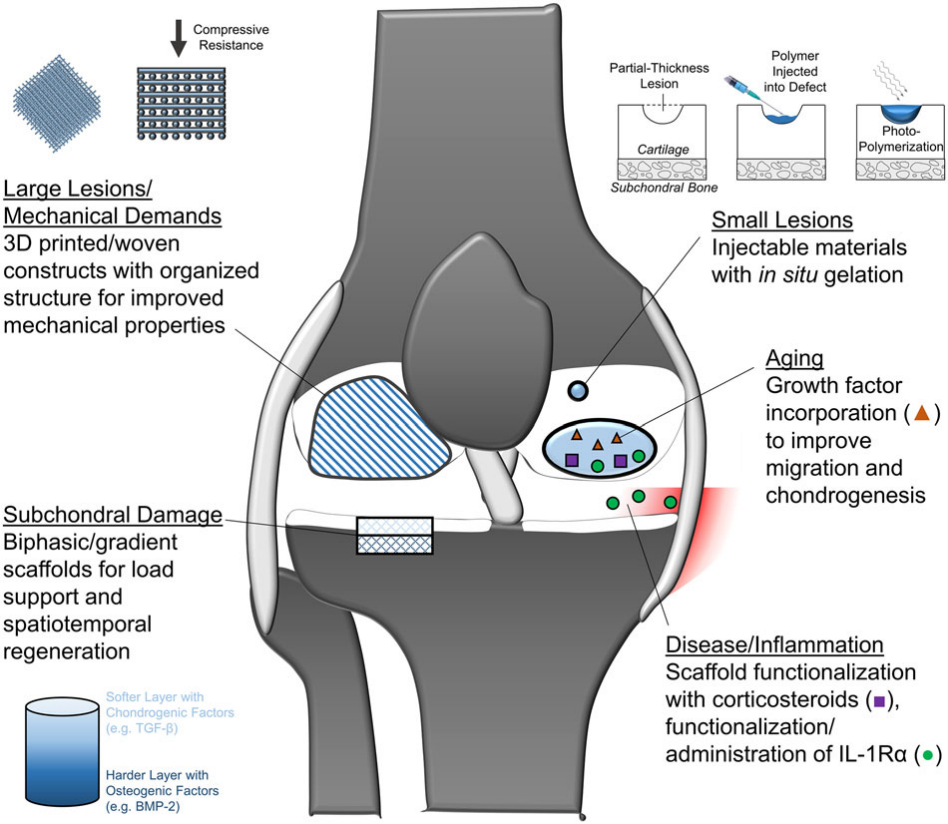
Summary figure showing emerging translational therapies for potential application in the ‘Red Knee’, including therapies that address large lesions, mechanical demands, subchondral damage, small lesions, aging, disease, and inflammation

Another important mechanical exclusion criterion in cartilage repair procedures is the presence of kissing lesions. Due to the continuous contact and articulation between two kissing implants, surface frictional and shear properties are of vital importance. Likewise, ligamentous instability and malalignment can exacerbate stresses parallel to the articular surface, increasing shear forces experienced by the implant and repair tissue.^91–93^ Therefore, along with bulk compressive mechanical properties, any cartilage replacement should minimize friction and maximize shear resistance to prevent wear or implant dislodgement. One study successfully increased lubricin (lubricating protein) concentration in superficial zone chondrocytes;^94^ this advance could be applied to cartilage scaffolds in order to reduce the coefficient of friction at the articulating surface. Another modality to decrease friction, that also provides shear resistance, is fabricating scaffolds with an aligned superficial zone.^95,96^ For example, Accardi and colleagues^97^ found that varying the alignment of electrospun nanofibers had a positive effect on implant shear properties. The group additionally varied electrospinning speed during fabrication to tune fiber organization through the scaffold depth to provide a shear-resistant superficial layer. These adaptations could be considered in knees requiring supplemental mechanical stability.

A previous attempt at cartilage repair is often an exclusion criterion for a subsequent cartilage regenerative procedure. This is driven by the likelihood of subchondral bone changes as a consequence of failed repair, particularly if the first attempt involved disruption of the tidemark for marrow recruitment. Without sufficient subchondral load support, subsequent cartilage treatment approaches may be predisposed to failure, and thus, the entire osteochondral unit must be considered in this cohort of patients. One potential intervention, currently used clinically, is osteochondral allograft transplantation (OATS).^98,99^ These grafts can provide symptom relief and success as a salvage procedure following failed cartilage repair. However, issues with graft survivability, disease transmission, and integration have motivated tissue engineers to develop newer composite scaffolds that guide localized regeneration of the cartilage and bone layers of an osteochondral unit.^100,101^ Clinically, the TruFit Plug (Smith & Nephew, San Antonio TX) is one of the only synthetic osteochondral scaffolds that has been evaluated in patients,^102,103^ with a subchondral phase containing calcium sulfate for bone regeneration and an articulating phase that relied on marrow stimulation for cartilage regeneration. While short-term results showed clinical improvement in patient MRI scores, improvement over conventional microfracture/OATS procedures has not been proven. In order to improve outcomes, biphasic scaffolds can be created with a softer upper layer containing chondrogenic factors (e.g., TGF-β, chondroitin sulfate) and a stiffer lower layer, often loaded with calcium phosphate, hydroxyapatite, or bone morphogenetic protein (BMP), to provide structural support of the above cartilage layer and to promote osteogenic tissue formation and boney integration.^53,104–106^ Bi-layered scaffolds derived from articular cartilage ECM and growth plate ECM can be used to regenerate osteochondral tissue with better recapitulation of the native architecture.^107^

To avoid the interfacial shear stress that are experienced between two such distinct layers, recent studies have developed gradient scaffolds, using the same growth factors and scaffold materials as a biphasic scaffold, but with a smooth transition between layers.^108,109^ For example, Di Luca et al.^109^ used a brush functionalization technique to create a gradient of TGF-β3, decreasing in concentration from the articulating surface to the subchondral region, and likewise created a reverse gradient of BMP-2. Other studies have utilized growth factor gradients, for example via microsphere incorporation.^108,110^ Furthermore, a transitional zone between cartilage and bone layers has been achieved via dispersion mixing with syringe pump systems, selective laser sintering, and pore shape gradients.^111–114^ The field of osteochondral tissue engineering has matured substantially and progress towards clinically applicable replacements will be vital for the large portion of the population currently excluded from clinical trials.

Patients with inflammation of the joint are often excluded from cartilage repair attempts given that catabolic cytokines and proteinases (MMPs) present in the synovial fluid degrade the native ECM, and would similarly degrade any implanted tissue, predisposing treatment failure. Interleukin-1β (IL-1β) and tumor necrosis factor-α (TNFα) are two important inflammatory cytokines that not only lead to cartilage matrix destruction, but also prevent chondrogenic differentiation of mesenchymal stem cells.^115^ These cytokines can be systemically upregulated, or produced by synoviocytes, chondrocytes, or meniscal cells.^116^ Also, co-morbidities, such as obesity, can elevate levels of pro-inflammatory cytokines, exacerbating the effects of a joint injury.^117^

To control the inflammatory environment and reduce proteolysis, intra-articular injection is clinically appealing. Such a treatment could be administered following a traumatic joint injury to prevent cartilage ECM proteolysis in the presence of pro-inflammatory cytokines (IL-1, IL-6, IL-8, and TNF-ɑ) and MMPs. To address this possibility, in a number of animal^118,119^ and human studies,^120^ high doses of anti-catabolic glucocorticoids, which inhibit the activation of MMPs^121^ and the expression inflammatory cytokines,^122^ have been administered. Dexamethasone (DEX) inhibits inflammation and cartilage damage by influencing both synoviocytes and chondrocytes.^119,123^ However, given the dynamic environment of the knee and the low residence time of small molecules in the synovial space, delivery and retention of molecules to positively impact cartilage regeneration before being cleared remains a challenge. Targeted intra-articular delivery of DEX can be achieved by using small positively charged nanoparticles bound to DEX to form electrostatic interactions between the cationic particle and the anionic cartilage matrix.^124^ Dendrimer-based nanocarriers were also recently shown to penetrate full-thickness cartilage explants, and be retained in a native joint environment.^125^ These nanoparticles may be powerful carriers for a glucocorticosteroid treatment.

In addition to localized glucocorticosteroid delivery, IL-1 receptor antagonist (IL-1Ra), a naturally occurring inhibitor of IL-1 activity, is another potential avenue for intra-articular anti-inflammatory therapeutics. There are currently multiple IL-1 targeting drugs on the market including Anakinra, a modified version of the human IL-Ra protein, and Canakinumab, a monoclonal antibody targeted at IL-1β. However, neither of these drugs has improved OA symptoms in human clinical trials to date, likely because of the dynamic joint environment.^126,127^ For either glucocorticosteroid or IL-1Ra delivery to be successful, the delivery mechanism (nanoparticles, scaffolds, etc.) is an extremely important design consideration. To improve the efficacy of IL-1Ra, researchers have tethered the protein to nanoscale block polymers to target cartilage.^128,129^ The IL-1Ra tethered polymeric nanoparticles were stable, non-toxic, and effective at blocking the IL-1 signaling pathway. In another study, IL-1Ra transgenes were incorporated into a woven scaffold.^61^ As a result, the stem cells in the scaffold released IL-1Ra in a sustained fashion over the course of 28 days in culture. Gene delivery of IL-1Ra through a regenerative scaffold or direct delivery of IL-1Ra via nanoparticle carriers are both promising options for cartilage repair in patients with post-surgical inflammation and other chronic inflammatory conditions.

## Conclusions and future outlook

Articular cartilage injuries are prevalent in a large portion of the population, and their incidence is only increasing. Cartilage regeneration technology has the potential to repair these lesions and prevent their progression to debilitating OA requiring total joint arthroplasty. Yet, the number of knee cartilage repair and regeneration procedures performed annually is dwarfed by the volume of total knee replacements. Current cartilage regeneration treatments approved for clinical use (MACI, AMIC) have shown satisfactory results in an optimal subset of patients with “green knees”. However, clinical trials assessing the efficacy of these techniques exclude the majority of the patient population with “red knees”, patients with large lesion size, high mechanical demands, older age, inflammation, infection, and other systemic diseases. Broadening the eligibility criteria of new trials would make the results more representative of the entire patient population. Following the footsteps of modern oncology research,^130^ cartilage regeneration trials should strive for inclusiveness. Exclusion criteria should have clear rational focusing on potential toxicities rather than efficacy concerns. Easier patient recruitment will allow analysis of larger and more representative populations, while subgroup analysis may help elucidate efficacy in a more selective “green knee” population. For example, including patients with common comorbidities would better highlight which conditions compromise cartilage restorative techniques and which do not. An alternative is to conduct secondary clinical trials in specific excluded populations, as is currently being done with MACI in pediatric patients.^131^

Translational research in the field of cartilage tissue engineering is working to improve treatment options (Fig. 5) to address comorbidities and reduce the number of excluded patients with cartilage lesions. Growth factors and other drugs released from scaffold materials can be used to recruit and rejuvenate cartilage progenitor cells in elderly patients. Scaffold material and fabrication technique can be tuned to provide greater surface area coverage and mechanical support. Targeted delivery of anti-inflammatory drugs may also improve scaffold integration and maturation in patients with inflammatory comorbidities.

Even more important than research in this field is the advancement from pre-clinical to clinical trials. The high cost, stringent regulatory oversight, and decades involved in the execution of rigorous clinical trials create barriers for advancement of this technology. Collaboration between large research groups and clinicians is key to the safe and successful progression of cartilage regeneration therapy, with the ultimate goal of providing all patients who have a cartilage injury with the opportunity for repair to conserve their joint rather than replace it.

### Reporting summary

Further information on experimental design is available in the Nature Research Reporting Summary linked to this paper.

## Supplementary information

Supplementary Material

Reporting Summary

## Data Availability

The inclusion and exclusion criteria data that support the findings of this study are available from ClinicalTrials.gov “https://clinicaltrials.gov/”. The synthesized raw data is also available upon request.

## Supplementary information

**Supplementary information** accompanies the paper on the *npj Regenerative Medicine* website (10.1038/s41536-019-0074-7).
