# Supplementary material for: Emerging therapies for cartilage regeneration in currently excluded ‘red knee’ populations
Source: NPJ Regen Med. 2019 May 30;4:12. doi: 10.1038/s41536-019-0074-7 (PMC6542813; doi:10.1038/s41536-019-0074-7)
Supplement: Supplementary file 1 — Supplementary Material [file 41536_2019_74_MOESM1_ESM.pdf]

Supplemental Table 1. Clinical Trials Systematically Selected for Extraction of Exclusion Criteria

1	**Confirmatory Study of NeoCart in Knee Cartilage Repair**
2	**Evaluation of an Acellular Osteochondral Graft for Cartilage Lesions ("EAGLE") European Post Market Study**
3	**Study on Efficacy and Safety of CaReSR-1S to Repair Cartilage Defects of the Knee**
4	**BiPhasic Cartilage Repair Implant (BiCRI) IDE Clinical Trial - Taiwan**
5	**HyaloFAST Trial for Repair of Articular Cartilage in the Knee**
6	**A Randomized Controlled Trial Comparing Chondro-Gide® to Microfracture Alone for Treatment of Knee Cartilage Defects**
7	**INSTRUCT for Repair of Knee Cartilage Defects**
8	**Trial Comparing BST-CarGel and Microfracture in Repair of Articular Cartilage Lesions in the Knee**
9	**DeNovo NT Longitudinal Data Collection (LDC) Knee Study**
10	**Post Market Study of DeNovo NT, Natural Tissue Graft**
11	**Evaluation of Safety and Exploratory Efficacy of CARTISTEM®, a Cell Therapy Product for Articular Cartilage Defects**
12	**Safety and Efficacy Study of HYTOP® in the Treatment of Focal Chondral Defects**
13	**ACI-C Versus AMIC. A Randomized Trial Comparing Two Methods for Repair of Cartilage Defects in the Knee**
14	**Study to Assess the Efficacy and Safety of Treatment of Articular Cartilage Lesions With CartiLife®**
15	**REcycled CartiLage Auto/Allo IMplantation**
16	**Tissue Engineered Nasal Cartilage for Regeneration of Articular Cartilage (Nose2Knee)**
17	**RCT of ChondroCelect® (in an ACI Procedure) vs Microfracture in theRepair of Cartilage Defects of the Knee**
18	**Clinical Trial for the Regeneration of Cartilage Lesions in the Knee (NosetoKnee2)**
19	**NOVOCART®3D for Treatment of Articular Cartilage of the Knee (N3D)**
20	**DeNovo NT Natural Tissue Graft Stratified Knee Study**
21	**Phase III Study to Evaluate Safety and Effectiveness of NOVOCART 3D Plus vs. Microfracture in Knee Cartilage Defects**
22	**NOVOCART® Inject Plus for Cartilage Defects of the Knee**
23	**Superiority of MACI® Versus Microfracture Treatment in Patients With Symptomatic Articular Cartilage Defects in the Knee**
24	**NuTech Affinity™ for the Treatment of Chondral Defects**
25	**Comparison of BioCartilage Versus Marrow Stimulating Procedure for Cartilage Defects of the Knee**
26	**Assessment of Efficacy and Safety of 3 Different Doses of co.Don Chondrosphere to Treat Large Cartilage Defects**
27	**Smith & Nephew's European Trufit Study**
28	**Agili-C™ Implant Performance Evaluation in the Repair of Cartilage and Osteochondral Defects**
29	**Study of the Treatment of Articular Repair (STAR)**
30	**Evaluation of the Agili-C Biphasic Implant in the Knee Joint**
31	**Efficacy and Safety Study of co.Don Chondrosphere to Treat Cartilage Defects**
32	**NeoCart Phase 2 Clinical Trial**
33	**Arthroscopic Autologous Chondrocyte Implantation Versus Microfractures**

Supplementary Methods

Creation of Figure 3

The search term "knee cartilage repair" was used on "clinicaltrials.com" on January 1st 2019. Studies that had been suspended, terminated, or withdrawn, as well as studies of unknown status, were excluded from the search, leaving 60 studies for review. Follow-up studies and studies using only intra-articular injections were also excluded. This yielded 33 clinical trials (Supplemental Table 1) that were used to extract inclusion and exclusion criteria. Criteria were tabulated in Microsoft Excel and the number of trials excluding a given criteria were summed to determine the percentage of trials excluding it. Percentage of exclusion was graphed by category and a threshold of 50% exclusion was used to identify criteria representing the "red knee".

Creation of Figure 4

To systematically review the mechanical properties of new cartilage scaffolds, a PubMed search was conducted in March 2018, with the search terms "cartilage", "scaffold", and "modulus". Manuscripts from January 2001 – January 2018 were included in this review. Of the 98 total studies, 79 studies (80.6%) presented a value from the mechanical evaluation. The values were further classified as a time-zero scaffold (red), a cultured construct (green), or regenerate tissue from in vivo studies (blue). Furthermore, the values were also divided into instantaneous (squares) and equilibrium (triangle) properties. The average modulus of the best group from these studies was plotted on a modulus versus year graph. In cases where data was presented in graphical format and not provided numerically within the text, data values were estimated.
